# Multicentric and Observational Study of Omalizumab for Chronic Spontaneous Urticaria in Real-Life in Colombia

**DOI:** 10.3389/falgy.2022.902344

**Published:** 2022-05-20

**Authors:** Elizabeth García-Gómez, Edgardo Chapman, María Beatriz García-Paba, Jaime Ocampo-Gómez, Eduardo Egea-Bermejo, Gloria Garavito-De Egea, Luis Fang, Mauricio Sarrazola, Jorge Mario Sánchez-Caraballo, Carlos Serrano-Reyes, Diana Lucia Silva-Espinosa, Dolly Vanessa Rojas-Mejía, Sergio M. Moreno

**Affiliations:** ^1^Departamento de Alergología, Fundación Santa Fé de Bogotá, Bogotá, Colombia; ^2^Facultad de Medicina, Universidad de los Andes, Bogotá, Colombia; ^3^Departamento de Alergología, Unidad Médico Quirúrgica de Otorrinolaringología (UNIMEQ-ORL), Bogotá, Colombia; ^4^Servicio de Alergología, Clínica de alergias de Colombia, Ibagué, Colombia; ^5^Departamento de Medicina, Universidad del Norte, Barranquilla, Colombia; ^6^Centro de Alergia e Inmunología Sarrazola, Clínica San José de Cúcuta, Cúcuta, Colombia; ^7^IPS Universitaria, Universidad de Antioquia, Medellín, Colombia; ^8^Unidad de Alergia, Fundación Valle de Lili, Cali, Colombia; ^9^Facultad Ciencias de la salud, Universidad ICESI, Cali, Colombia

**Keywords:** urticaria, angioedema, Omalizumab, inducible, antihistamines

## Abstract

**Background:**

Although chronic urticaria (CU) is a common, cause of medical consulting both in general practitioners and allergist specialists worldwide, there is little information about its behavior and management in Latin America. Currently, national and international guidelines recommend using Omalizumab for cases refractory to management with antihistamines. Despite advances in the knowledge of Omalizumab for the management of CU, although there are few studies in underdeveloped countries, there are many studies evaluating the impact of Omalizumab treatment. There is not clinical information related with CSU-Omalizumab in patient settled in the Caribbean area. This research aims to evaluate the management of CU with Omalizumab in a real-life scenario in Colombia.

**Methodology:**

We conducted an observational, descriptive, and retrospective study with patient recruitment between 2014 and 2017 of individuals diagnosed with Chronic Urticaria (CU) treating allergology specialists in five Colombian cities. We included patients with CU who failed to achieve disease control after treatment for 4 weeks with fourfold doses of second-generation H1-antihistamines, as recommended by the EAACI/GA^2^LEN/EDF/WAO guidelines and who received treatment with Omalizumab.

**Results:**

We included 123 patients, 73.1% (*n* = 90) were women. The mean age was 47.1 years (Standard Deviation, SD: 16.2). The median of the total months of disease evolution was 30 (IQR = 13–58). 81.3 % (*n* = 100) of patients were diagnosed with chronic spontaneous urticarial (CSU). 4.8% (*n* = 6) had inducible CU (CIndU), and 13.8% (*n* = 17) reported mixed urticaria (spontaneous CU with at least one inducible component). Regarding emotional factors, 34.9% (*n* = 43) of subjects indicated anxiety symptoms, 34.1% (*n* = 42) had exacerbations associated with stress, and 14.6% (*n* = 18) manifested episodes of sadness. The percentage of patients with CSU controlled according to medical criteria at 3 months with Omalizumab were 80% (*n* = 80/100) and at 6 months 87% (*n* = 87/100). The frequency of adverse events was 29.2% (*n* = 36), with headache being the most frequent adverse event.

**Conclusions:**

This real-life study with Omalizumab at CU describes percentages of effectiveness and safety similar to those observed in pivotal and real-life studies conducted in other regions around the world.

## Introduction

Urticaria is a disease characterized by the sudden appearance of hives, pruritus. Angioedema appears in about 40% of patients. Secondary to releasing inflammatory mediators such as histamine by mast cells present in the skin. It is chronic when the symptoms occur for at least 6 weeks (1). Chronic Urticaria (CU) is classified in Chronic Spontaneous Urticaria (CSU) and Chronic Inducible Urticaria (CIndU) (2). CSU was previously known as idiopathic urticarial.The triggering factor is not identified in most case of CSU, and the onset of symptoms is unpredictable (3). In CIndU, generally, a physical stimulus is consistently recognized that initiates lesions (4).

The exact prevalence of CU is currently unknown. Zuberbier et al. (5) reported a lifetime prevalence of 1.8% (95% CI 1.4–2.3%) in German adults surveyed over 3 years. A recent study with 3,538,540 Germans during 2017 reported 17,524 patients (0.5%) diagnosed with CU; chronic spontaneous urticaria (CSU: 71.2%), chronic inducible urticaria (CIndU: 19.7%), and CSU+CIndU [9.1%; (6)]. In another study, the prevalence in children was 0.1–0.3% (7). About CU duration is variable; however, it has been reported 6 to 12 weeks in 52.8% of patients, 3–6 months in 18.7%, 7–12 months in 9.4%, 1–5 years in 8.7%, and more than 5 years in 11.3% (8).

Several etiological factors have been associated with CU, including autoimmune diseases, allergens, pseudo-allergens, and infections, but it is challenging to identify the specific trigger in most patients (9, 10). Autoimmune and inducible conditions may be more resistant to treatment and have a prolonged course (11).

CU affects the quality of life of those who suffer from it, causing a significant commitment to work and school activities, anxiety, and depression, with negative consequences on health services and society (12–14).

One of the treatments used in clinical practice for disease control is Omalizumab. A humanized anti-IgE monoclonal antibody approved as an adjunct treatment for CU in people over 12 years of age with inadequate responses to antihistamine anti-H1 treatment (1, 9, 15–17).

In underdeveloped countries and the Latin American region, little is known about the clinical features of CU in patients receiving Omalizumab (18–22). This study aims to describe the characteristics and clinical response to treatment with Omalizumab in patients with CU in real life in Colombia.

## Materials and Methods

### Study Design

This is an observational, descriptive, and retrospective study. Between the years 2014 and 2017, we recruited patients that meet the following criteria: (i) older than 12 years of age; (ii) clinical diagnosis of CU; (iii) disease duration >6 weeks; (iv) being under symptomatic pharmacological treatment according to EAACI/GA^2^LEN/EDF/WAO guidelines (23); (v) failed disease control and be refractory to fourfold doses of second-generation H1-antihistamine therapy after 4 weeks; and (vi) received Omalizumab therapy. Patients weighing <20 kg, known hypersensitivity to Omalizumab or pregnancy were excluded. We retrieved clinical information on these patients from clinical allergologist in five Colombian cities (Bogotá, Cali, Medellín, Barranquilla, and Cúcuta).

This study was endorsed by the Ethics and Biomedical Research Committees of Fundación Santa Fe de Bogotá (Bogotá—Colombia), Fundación Valle del Lili (Cali—Colombia), and Fundación Universidad del Norte (Barranquilla—Colombia). All participants gave their informed consent before being included in the study.

### Study Variables

We reviewed the medical record looking for sociodemographic variables, family history of autoimmune disease, and atopic state of the patient. We also took into account the reports of laboratory test such as antinuclear antibodies (ANA), anti-DNA antibodies, anti-phospholipid antibodies, rheumatoid factor, anti-thyroperoxidase antibody (anti-TPO), anti-myeloperoxidase antibodies (anti-MPO), autologous serum skin test (ASST), rapid plasma reagin test (RPR), and skin biopsy. We consider the criteria of the EAACI/GA^2^LEN/EDF/WAO guidelines for definition, classification, diagnosis, and management of urticaria (23).

In addition, we recovered from medical records information about the activity of the disease and emotional alterations associated with the diagnosis of CU registered prior to treatment with the clinical allergologist researchers in this study. We reviewed the time in years from the diagnosis of urticaria to its implementation, dose, duration, outcomes, and adverse events of treatment about Omalizumab therapy.

### Statistical Analysis

The present study is descriptive, with no hypothesis or comparison/intervention under consideration. The variables were measured standardized, and these do not have subjectivity regarding their occurrence. Therefore, we do not consider a significant risk of bias in this study. A complete descriptive analysis was performed with absolute and relative frequencies for the qualitative variables and parameters of central tendency together with the maximum and minimum values for quantitative variables according to the nature of the variable. Additionally, a stratified analysis was performed by age group, sex, and socioeconomic stratum of the study subjects. For statistical analysis, we used Stata v14 software.

## Results

### Demographic and Clinical Characteristics of Patients With CU

One hundred twenty-three patients diagnosed with CU were included in the analysis. The mean age was 47.1 ± 16.2 years. 73.1% (*n* = 90) of the patients were women. The duration of the disease at the time of inclusion in the study showed a median of 30 months (IQR 13–58 months) (Table 1).

**Table 1:** Demographic and clinical characteristics of patients with chronic urticaria.

	***n*** **=** **123**	
	***N***	**%**
Sex		
Female	90	73.1
Age—Mean (SD)	47.1 (16.2)	
Months of evolution of the UC—Median (p25–p75)	30 (13–58)	
Type of chronic urticaria		
Spontaneous	100	81.3
Mixed	17	13.8
Inducible	6	4.8
Angioedema	49	39,8
Comorbidities		
Respiratory allergy	20	16.2
Drug allergy	7	5.6
Atopic dermatitis	4	3.2
Other allergic disease	3	2.4
Systemic lupus erythematosus	1	0.8
Autoimmune thyroiditis	9	7.3
Rheumatoid arthritis	2	1.6
Sjogren's syndrome	2	1.6
Vitiligo	1	0.8

*SD, standard deviation*.

81.3% (*n* = 100) of patients were diagnosed with chronic spontaneous urticarial—CSU. 4.8% (*n* = 6) of the individual showed chronic inducible urticaria (CIndU). Heat (*n* = 3), cold (*n* = 2), and cholinergic urticaria (*n* = 1) were identified as triggering factors. 13.8% (*n* = 17) of patients reported mixed urticaria, i.e., CSU with at least one inducible component; dermographism being the most frequent (*n* = 12) (Table 1).

Angioedema was observed in 39.8% (*n* = 49) of study subjects. We observed respiratory allergies (16.2%; *n* = 20), autoimmune thyroiditis (7.32%; *n* = 9), drug allergy (5.6%; *n* = 7), among other comorbidities in these patients (Table 1).

### Paraclinical Findings Observed in Patients With CU

Antinuclear antibodies (anti-ANA) were the most requested laboratory test (65%; *n* = 80); however, it only showed 15% (*n* = 12/80) positivity among patients tested. Other tests of markers related to autoimmunity were also requested. In contrast, the autologous serum test (AST) was performed on only 13% (*n* = 16) of patients; however, it showed a positivity of 25% (*n* = 4/16; Table 2). Regarding serum total IgE concentrations, this test was performed on 34.1% (*n* = 42) of patients showing an average of 244.7 IU/ml (SD: 397 IU/ml), with a minimum value of 6.5 IU/ml and a maximum of 2,500 IU/ml.

**Table 2:** Paraclinical tests recorded in the study.

	**Tests performed** **(*n*%)**	**Positive tests (n)**	**Positivity (%)**
Antinuclear antibodies (ANA)	80 (65%)	12	15.0
Anti-DNA antibodies	57 (46.3%)	1	1.7
Anti-phospholipids antibodies	40 (32.5%)	2	5.0
Rapid plasma reagin test (RPR)	31 (25.2%)	3	9.6
Rheumatoid factor	48 (39%)	3	6.2
Anti-thyroperoxidase antibody (anti-TPO)	61 (49.6%)	9	14.7
Anti-myeloperoxidase antibodies (anti-MPO)	45 (36.6%)	5	11.1
Autologous serum skin test (ASST)	16 (13%)	4	25.0
Biopsy	15 (12.2%)	4	26.6

### Disease Activity and Emotional Alterations Associated With the Diagnosis of CU

The disease activity was measured and recorded in the medical records of only 89.4% (*n* = 110) of the subjects; in 56.3% (*n* = 62/110) of patients, the activity of the disease was measured by clinical interview. The implementation of a “*patient-reported outcome measures”* (PROMs) questionnaire was observed in 57.3% (*n* = 63/110) of subjects employing the urticarial control test (UCT) questionnaire.

Before first interview with the allergology specialist, and previously to starting treatment with Omalizumab, 82.1% (*n* = 101) of patients were being treated according to the EAACI/GA^2^LEN/EDF/WAO guideline (23). 16.3% (*n* = 20) of participants had prescribed at least two drugs combination. 45.5% (*n* = 56) of subjects were treated with first-line of treatment (standard doses of second-generation H1-antihistamines for 2 weeks); however, symptoms persisted and the dose was increased four-fold for 4 weeks (second-line of treatment). 21.9% (*n* = 27) of patients were treated with fourfold dose of 2nd generation H1-antihistamines (second-line of treatment); however, symptoms persisted after 4 weeks of treatment. We observed that 4.88% (*n* = 6) of patients were being treated with a therapy that combined fourfold dose of 2nd generation H1-antihistamines accompanied with short course of oral corticosteroids, antileukotrienes and immunomodulators (Ciclosporin) drugs (Third-line of treatment); nevertheless, symptoms persisted.

Chronic urticaria is a disease that affects the quality of life of those who suffer from it. Of the total number of subjects evaluated, 34.9% (*n* = 43) indicated anxiety symptoms, 34.1% (*n* = 42) had exacerbations of symptoms associated with stress, 14.6% (*n* = 18) of the subjects described episodes of sadness, up to suicidal ideation in 0.8% (Table 3).

**Table 3:** Emotional alterations associated with the diagnosis of CU.

	***n*: 123**	**%**
Anxiety	43	34.9
Stress-associated exacerbations	42	34.1
Sadness	18	14.6
Sleep disturbance	17	13.8
ideas of handicap	3	2.4
Suicidal thoughts	1	0.8

### Findings Observed During Treatment With Omalizumab

All patients received Omalizumab for being refractory to four-fold doses of second-generation H1-antihistamine therapy after 4 weeks. 37.4% (*n* = 46) of patients received Omalizumab between 6 and 12 months. 86.9% (*n* = 107) received a dose of 300 mg every 4 weeks. 6.5% (*n* = 8) received 150 mg of Omalizumab, and 6.5% (*n* = 8) of patients were treated with 600 mg of Omalizumab every 4 weeks.

**Figure 1:**
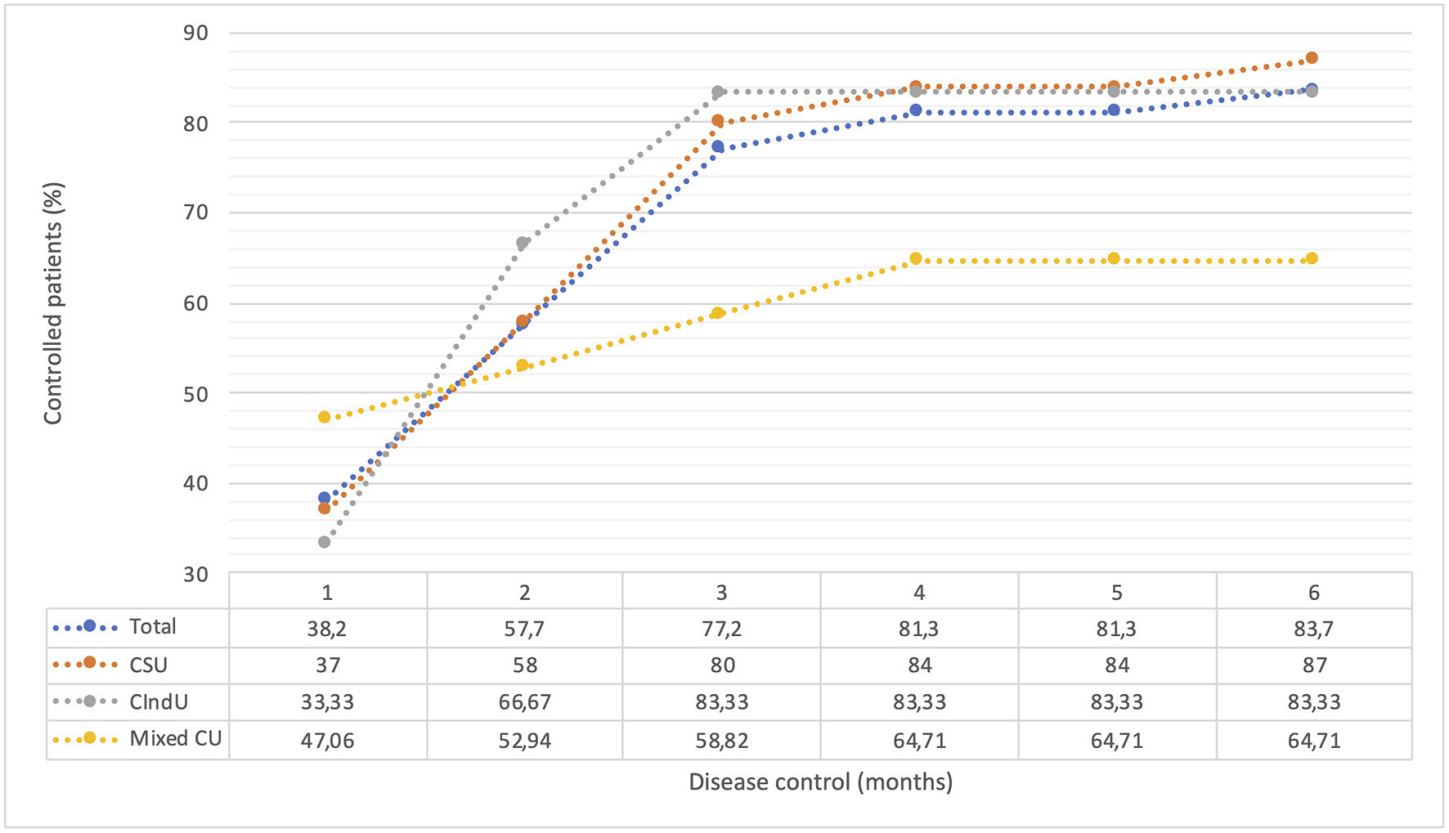
Control of the disease with the use of Omalizumab stratified by type of Chronic Urticaria. Disease control was defined according to the presence of symptoms of urticaria in last 4 weeks of treatment with Omalizumab. Some patients were evaluated using the UCT; those patient with a UCT-score ≥12 was considered as “well-controlled disease” (56%; *n* = 69), while others were evaluated using the clinical interview employing questions comparable to the UCT (57%; *n* = 70). CU, chronic urticaria; CSU, chronic spontaneous urticaria; CIndU, chronic inducible urticaria; UCT, urticaria control test.

Forty-seven (38.21%; *n* = 47) patients showed disease control after the 1st month of treatment with Omalizumab. This quantity increased to 83.74% (*n* = 103) at 6 months of therapy. Participants with Chronic Spontaneous Urticaria presented the highest percentage of improvement (87%; *n* = 87/100) at 6 months of treatment. A very similar finding in patients with CIndU who reported an improvement of 83.3% (*n* = 5/6) from the third month of treatment. In contrast, only 64.7% (*n* = 11/17) of patients with mixed urticaria reported improvement at 6 months of Omalizumab therapy (Figure 1).

Regarding the safety of Omalizumab, 29.2% (*n* = 36) of patients reported at least one adverse event associated with the drug; However, this was not a reason to discontinue treatment. Headache being the most frequent (36.1%; *n* = 13/36), followed by myalgia 19.4% (*n* = 7/36), local pain and inflammation at the administration site (11.1%; *n* = 4), arthralgias (8.3%; *n* = 3), among other side effects (Table 4).

**Table 4:** Adverse events reported during omalizumab treatment.

**Adverse events**	***n*: 36**	**%**
Headaches	13	36.1%
Myalgias	7	19.4%
Arthralgias	3	8.3%
Local pain and inflammation	4	11.1%
Other AEs*	9	25%

*Adverse events.

## Discussion

This is the first study conducted in Colombia that sought to characterize CU patients managed with Omalizumab providing data on the response and safety of this drug in real-life. Of the patients evaluated, a clear predominance of the female gender was found, coinciding with what was found in previous studies (8, 18, 22, 24). The average age was 47.3 years, similar to that reported by Gaig et al. in a large population study conducted in Spain, where a higher incidence of this disease was found between 25 and 55 years (8). As in other published studies (18), patients with CSU received Omalizumab more frequently than mixed and inducible forms (25, 26). Dermographism was the most frequent physical trigger. Like other reports, factors such as cold, heat, pressure, and cholinergic urticaria were rarer (27–29).

Angioedema is considered an unfavorable prognostic factor for CU (30). Our study observed a prevalence of 39.8% (*n* = 49), comparable data in a range of 38–54% in contrast to other clinical reports (31, 32). On the other hand, mental comorbidities have been associated with CU (12, 33, 34), a finding corroborated in our study. More than a third of the patients evaluated reported symptoms related to anxiety and exacerbations associated with stress.

There are currently no reliable biomarkers to measure disease activity in CU. However, patient-reported outcome measures (PROMs) are of great importance (13, 25, 35). PROMs allow the evaluation of various aspects of the disease, such as activity, severity, and control (35). However, a systematic review of evidence in the “real life” by Bernstein et al. (36) showed low use, with UAS7 and UCT being used in only 28.6 and 3.6% of studies, respectively. In our study, 89.4% (*n* = 110) of the subjects, the activity of the disease were evaluated by the clinical interview and the UCT being the most used PROM questionnaire, reported for use in more than half of the patients.

Current guidelines recommend limiting routine laboratory tests for CSU, C-reactive protein (CRP), and complete blood count (CBC) being basic tests (1, 2, 9). In contrast, other paraclinical tests should be requested based on medical records and physical examination, particularly patients with the longer-term or uncontrolled disease (1). In our study, the most requested laboratory test was the autoantibodies anti-ANA (65%); remember the association between CU and significant autoimmune diseases (37, 38). Regarding serum concentrations of total IgE, an average of 244.7 IU/ml (SD = 397 IU/ml) was observed, similar to that reported by Saini et al. with 215.3 IU/ml (SD = 431.6 IU/ml). Patients with CSU have lower levels of total IgE in contrast to patients with asthma (39). There is little evidence to support the association between serum IgE levels and CSU; however, recent studies show that CU's low IgE levels serve as a hyporesponsive marker to Omalizumab (40–43).

In this study, most patients were treated according to the EAACI/GA^2^LEN/EDF/WAO guideline (23). This guideline recommends using second-generation H1 antihistamines as the first line of treatment. In cases where disease control is not achieved, it suggests increasing the dose of this medication up to four doses compared to the standard dose. We found that 45.5% (*n* = 56) of patients received second-generation anti-H1 at standard and quadruple doses (21.9%; *n* = 27) during the first consultation with the clinical allergologist. The use of immunosuppressive drugs before Omalizumab was lower than in the GLACIAL study (4.8 vs. 9.5%), as were systemic corticosteroids (16.8 vs. 57.9%) and antileukotrienes (19.2 vs. 57.5%) (44). This reduction, especially in immunosuppressants and corticosteroids, is essential because of the adverse effects commonly reported with these drugs.

Regarding the use of Omalizumab, in this study, most patients (86.9%; *n* = 107) received a dose of 300 mg every 4 weeks, similar to the studies that report the efficacy of the drug in CU administered in this therapeutic regimen (45, 46). Only 6.5% (*n* = 8) of patients were treated with 600 mg of Omalizumab every 4 weeks. More evidence is needed to show whether the 600 mg dose is more effective than 300 mg (39). In addition, the standard dose of Omalizumab may be sufficient for disease control, independent of weight and serum total IgE concentration. Omalizumab acts directly on mast cell/basophil reactivity, which would reduce the formation of hives relatively quickly rather than requiring a long-term change in serum IgE levels to a steady-state necessary for asthma control (40, 42).

Eighty-seven percent (87%; *n* = 87/100) of the patients with CSU in this study presented better disease control using Omalizumab. This result is higher than that reported in the meta-analysis by Rubini et al. of seven controlled-randomized clinical trials, where 1,312 cases showed a response rate of 36% (47). Although, our findings are closer to others “real life” studies that describe better CU remission rates with Omalizumab (18, 26, 27); a possible explanation for the observed findings in clinical trials is the use of questionnaire that measure disease activity such as the UCT or the UAS, which in our study were not constantly used. Furthermore, treatment response criteria are less stringent in real-life studies. Although comparing results between different studies is difficult because populations, dosing regimens, assessment scores, and response definitions differ from study to study. Our study reports a 38.2% (*n* = 47) of rate of early responders to Omalizumab. Similar to results of the ASTERIA I clinical study (37% of early responders) (46), although slightly lower than that reported in ASTERIA II (51%; 39). Cherrez-Ojeda et al. showed that 45.5% of the patients responded to the drug in the 1st month of treatment in their Latin American real-life study (18). This previous study reported a median duration of treatment with Omalizumab was 7.67 months, within the range found in our investigation of 6 and 12 months, as the most frequent duration in which patients received the drug.

One of the advantages of this study is the inclusion of patients with other forms of chronic urticaria other than spontaneous. We evaluated six patients (4.8%; *n* = 6) with Chronic Inducible Urticaria (CIndU), where we showed the effectiveness of Omalizumab. Although Omalizumab in these cases is “off-label,” the Colombian health system allows the formulation of this drug under the name of Chronic Urticaria without taking into account its subtype.

The use of Omalizumab in the treatment of CIndU continues to be an “off-label” use. However, there is mounting evidence of clinical reports which used this therapeutic alternative. Chicharro and Rodríguez de Argila (48) compiled case reports and case series describing the use of Omalizumab to treat CIndU, concluding that Omalizumab is a potentially effective and safe alternative in the treatment of some cases of CIndU. However, more studies are needed to evaluate the safety and efficacy of drugs such as Omalizumab, for the treatment of this condition, with a more significant number of patients and a solid prospective, double-blind, placebo-controlled methodological design.

Regarding the safety of Omalizumab, no observed severe adverse effects in this research. Headache was the most frequent adverse event, followed by myalgia, and arthralgias; adverse events equivalent to another clinical study in CU (47). Although headache is the most common neurological side effect and also noted that omalizumab led to musculoskeletal disturbances including low back pain, arthralgia, pain in the extremities, and myalgia; the mechanisms underlying the development of adverse events are unknown, these come to be given by the condition of each individual. In our study, a high frequency of the events described above was observed in relation to other studies (49). Only four patients reported local pain and inflammation at the administration site (3.2%; *n* = 4), none of the patients met criteria for anaphylaxis. Data from post-marketing studies have shown that these reactions are infrequent and anaphylaxis very rare (0.09%) (50).

We consider that the main limitations of this study include its retrospective design because we obtain information directly from patients or their medical records; this can lead to information bias. Another limitation is the small number of patients with CIndU, which does not allow conclusions about the effectiveness of Omalizumab on this condition. It was not possible to evaluate the quality of life as reported by other studies; instruments such as the Dermatology Life Quality Index (DLQI) or chronic urticaria quality of life questionnaire (CU-QoL) are little used in the Colombian health system. Although the preferred guideline for allergists participating in this study was the EAACI/GA^2^LEN/EDF/WAO guideline (1), it is not common to use the UAS7 to measure disease activity. Therefore the activity of the disease was measured according to clinical interview and the Urticarial Control Test (UCT) questionnaire.

## Conclusions

This real-life study with Omalizumab in CU describes effectiveness and safety percentages similar to those observed in pivotal and real-life studies conducted in other regions. It confirms the presence of emotional disorders such as anxiety, depression, and sleep disturbances in patients suffering from the disease. Likewise, we corroborated the need to use objective evaluation tools for the activity and control of the disease. It is necessary for prospective clinical studies with a significant number of patients with different subtypes of CU to validate the efficacy and safety of the Omalizumab in the long term and in “real life” conditions. As well as individualize each patient through predictive factors of response to treatment to offer the best therapist option for their disease.

## Data Availability Statement

The raw data supporting the conclusions of this article will be made available by the authors, without undue reservation.

## Ethics Statement

The studies involving human participants were reviewed and approved by the Ethics Committees of Fundación Santa Fé de Bogotá (Colombia), Fundación Valle de Lili (Colombia), and Universidad del Norte (Colombia). The patients/participants provided their written informed consent to participate in this study.

## Author Contributions

All authors listed have made a substantial, direct, and intellectual contribution to the work and approved it for publication.

## Funding

The participating institutions financed the development of this study.

## Conflict of Interest

The authors declare that the research was conducted in the absence of any commercial or financial relationships that could be construed as a potential conflict of interest.

## Publisher's Note

All claims expressed in this article are solely those of the authors and do not necessarily represent those of their affiliated organizations, or those of the publisher, the editors and the reviewers. Any product that may be evaluated in this article, or claim that may be made by its manufacturer, is not guaranteed or endorsed by the publisher.
